# Penetrance of colorectal cancer among *MLH1*/*MSH2 *carriers participating in the colorectal cancer familial registry in Ontario

**DOI:** 10.1186/1897-4287-7-14

**Published:** 2009-08-23

**Authors:** Yun-Hee Choi, Michelle Cotterchio, Gail McKeown-Eyssen, Monga Neerav, Bharati Bapat, Kevin Boyd, Steven Gallinger, John McLaughlin, Melyssa Aronson, Laurent Briollais

**Affiliations:** 1 Prosserman Centre for Health Research, Samuel Lunenfeld Research Institute, Toronto, Canada; 2 Department of Epidemiology and Biostatistics, University of Western Ontario, London, Canada; 3 Division of Preventive Oncology, Cancer Care Ontario, Toronto, Canada; 4 Division of Population Studies and Surveillance, Cancer Care Ontario, Toronto, Canada; 5 Dalla Lana School of Public Health, University of Toronto, Toronto, Canada; 6 Department of Nutritional Sciences, University of Toronto, Toronto, Canada; 7 Fred A. Litwin Centre for Cancer Genetic, Samuel Lunenfeld Research Institute, Toronto, Canada; 8 Laboratory of Medicine and Pathobiology, Mount Sinai Hospital, Toronto, Canada; 9 Department of Surgery, University of Toronto, Toronto, Canada; 10 Ontario Familial Colon Cancer Registry, Ontario Cancer Genetics Network, Toronto, Canada; 11 Dr. Zane Cohen Digestive Disease Clinical Research Centre, Mount Sinai Hospital, Toronto, Canada

## Abstract

**Background:**

Several DNA mismatch repair (MMR) genes, responsible for the majority of Lynch Syndrome cancers, have been identified, predominantly *MLH1 *and *MSH2*, but the risk associated with these mutations is still not well established. The aim of this study is to provide population-based estimates of the risks of colorectal cancer (CRC) by gender and mutation type from the Ontario population.

**Methods:**

We analyzed 32 families segregating MMR mutations selected from the Ontario Familial Colorectal Cancer Registry and including 199 first-degree and 421 second-degree relatives. The cumulative risks were estimated using a modified segregation-based approach, which allows correction for the ascertainment of the Lynch Syndrome families and permits account to be taken for missing genotype information.

**Results:**

The risks of developing CRC by age 70 were 60% and 47% among men and women carriers of any MMR mutation, respectively. Among *MLH1 *mutation carriers, males had significantly higher risks than females at all ages (67% vs. 35% by age 70, p-value = 0.02), while the risks were similar in *MSH2 *carriers (about 54%). The relative risk associated with *MLH1 *was almost constant with age (hazard ratio (HR) varied between 5.5-5.1 over age 30–70), while the HR for *MSH2 *decreased with age (from 13.1 at age 30 to 5.4 at age 70).

**Conclusion:**

This study provides a unique population-based study of CRC risks among *MSH2*/*MLH1 *mutation carriers in a Canadian population and can help to better define and understand the patterns of risks among members of Lynch Syndrome families.

## Introduction

Lynch Syndrome, also referred to as Hereditary non-polyposis colorectal cancer (HNPCC) is an autosomal dominant condition which predisposes carriers to both colorectal and extra-colonic cancers [1]. Several DNA mismatch repair (MMR) genes responsible for the majority of Lynch Syndrome cancers have been identified, predominantly *MLH1 *and *MSH2 *[2]. An understanding of risk associated with MMR mutations is important to assist in decisions about prophylactic surgery, annual screening and chemoprevention and to help allay psychological distress related to the uncertainty of colorectal cancer (CRC) predisposition among Lynch Syndrome family members [1].

Despite this importance, only a small number of studies provided penetrance estimates for these gene mutations. In addition, most of them studied populations from Finland, Scotland, the Netherlands and Nebraska [3-6] and it is unlikely that they are generalizable to the Canadian population as genetic mutations, lifestyle and environmental factors may differ. Moreover, most previous studies focused on families with a very strong history of cancers associated with the Lynch Syndrome and the derived penetrance estimates are only valid for families with comparable family history. Our study provides population-based estimates of penetrance by age, sex and mutated gene for CRC cases identified through the Ontario Familial Colorectal Cancer Registry (OFCCR) [7] and selected to be representative of families likely to harbour MMR mutations. We also used an ascertainment-corrected segregation approach to correct for the specific ascertainment and missing mutation information. Therefore, our analyses reduce the chance for biases and provide penetrance estimates that are applicable to a larger population of CRC cases harbouring MMR mutations.

## Materials and methods

### Study population

The OFCCR is one of six international registries established by the National Cancer Institute (NCI) as a resource for the study of CRC [7]. The OFCCR used the population-based Ontario Cancer Registry (OCR) to identify incident CRC cases (probands), aged 20–74, diagnosed July 1997 to July 2000. With physician consent, colorectal cases were contacted by a letter describing the study and requesting completion of a family history questionnaire (FHQ) which ascertained birth, death and disease history for first- and second-degree relatives. This information was used by genetic counselors to produce family pedigrees and to classify families as either 1) Amsterdam 1, 2) Familial Risk which included early-onset CRC and/or extra-colonic cancer in proband or relative, or family history of multiple CRC and/or extra-colonic cancers, 3) Sporadic/low risk with no family history of CRC [7]. Probands from Amsterdam 1 and Familial Risk families were interviewed by counselors to clarify information regarding all first-degree relatives, complete the pedigree to include all second-degree relatives, and ascertain full cancer histories. A randomized sample of 25% of the sporadic/low risk cases was also studied but because the prevalence of MMR germline mutations is very low in the general population, around 1:3139 [8], this group was not further considered in the penetrance study. Indeed, only one individual in this group was found mutation carrier after tumor analysis and germline testing.

Whenever possible, probands' tumours were first screened for microsatellite instability (MSI) and immunohistochemistry (IHC). Germline mutation analysis of *MSH2, MLH1 *and/or *MSH6 *genes was initiated in probands based on MSI-high and/or IHC deficient tumours results. Germline screening of a specific MMR gene was guided by IHC deficiency for a specific MMR protein. Germline mutations were also assessed among probands with MSI-low or microsatellite stable (MSS) tumours if their tumours exhibited MMR protein deficiency when assessed by IHC [9]. Probands were also screened for germline mutations in *MLH1 *and *MSH2 *when the tumours were MSI-high and IHC intact, or where tumours were not available for probands in Amsterdam 1 families. Testing for MMR genes involved sequence analysis (*MSH2, MLH1, MSH6*) and Multiplex Ligation-dependent Probe Amplification (MLPA) (*MSH2*, *MLH1*) of genomic DNA obtained from blood samples [9].

For each proband found to carry an MMR mutation, all first- and second-degree relatives on the "at-risk side" of the proband's pedigree were considered to be eligible for the present study. Blood samples from these relatives were obtained in two stages. First, the OFCCR had requested blood samples from all probands' first-degree relatives, together with all second-degree relatives who had had cancer or who were a first-degree relative of a family member with cancer, and who resided in North America, most European countries, or the Caribbean. Second, to ensure as many kin as possible were ascertained for the present study, we requested blood from all probands' second-degree relatives who had not previously been contacted by the OFCCR. Inclusion of these additional second-degree relatives was important to avoid over representation of relatives with a cancer diagnosis and resultant upwardly biased risk estimates.

Mutations in the MMR genes were ascertained from genomic DNA for all participants from whom blood samples could be obtained. For deceased relatives, obligate gene mutation carriers were defined as persons with a descendent with a MMR gene mutation. Therefore, the final retrospective study cohort included all living *MSH2, MLH1 *and *MSH6 *mutation carriers, and all deceased obligate gene mutation carriers. There were 446 probands eligible for our study (i.e., with blood sample available, with consent to contact their relatives, satisfying the Amsterdam 1 or other familial risk criteria, see above). Of these, 358 were tested for IHC (80%), 346 for MSI (77%), and 379 for either IHC or MSI (85%). Our final cohort included 32 probands found to carry a MMR mutation and their relatives. There were 27 probands from Amsterdam 1 families and 5 from other familial risk families. The relatives consisted of 199 first-degree and 421 second-degree relatives, from whom 56 and 41, respectively, were CRC cases (see Table 1). The number of blood samples available was 71 and 56, respectively, in first- and second-degree relatives from whom 38 and 22 were tested mutation positive (see Table 1). Survival analyses with the classical Kaplan-Meier estimator [10] were carried out on the sample of individuals with blood available. The modified segregation-based approach (see methods section) was able to use the full cohort of individuals (i.e. with or without blood available) but age at diagnosis or at examination was required for the analysis. This was available for 506 individuals of the 622 recruited.

**Table 1:** Description of MMR mutation-carrying participants – mutation information and counts of kin (family size), kin with blood, colorectal cancer (CRC) amongst kin, and mutation carriers amongst kin by relative degree

			**No. of kin reported**		**No. of kin with CRC reported**		**No. of kin with blood**		**No. of mutation carriers**	
			
**Gene**	**Germline Mutation**	**Mutation Consequence**	**1^st^**	**2^nd^**	**1^st^**	**2^nd^**	**1^st^**	**2^nd^**	**1^st^**	**2^nd^**
*MLH1*	c.1852_1853 AA>GC	p.Lys618Ala	4	16	2	0	2	5	0	0
	c.1689_1690insA	p.Ile563IlefsX4	3	12	1	2	2	0	0	0
	c.1732_1896del	Exon 16 del	6	11	2	0	2	4	1	3
	c.116+5 G>C	Splice-site defect	7	13	1	1	2	0	1	0
	c.793C>T	p.Arg265Cys	10	23	2	5	1	6	1	1
	c.793C>T	p.Arg265Cys	7	25	1	5	2	5	1	0
	c.346delA	p.Thr116GlnfsX20	2	5	1	2	0	0	0	0
	c.731G>A	p.Gly244Asp	6	5	2	0	5	0	5	0
	c.790+2T>C	Splice-site defect	9	19	3	1	6	7	3	3
	c.298C>T	p.Arg100X	7	6	2	0	1	0	0	0
	c.1975C>T	p.Arg659X	3	5	2	3	3	0	0	0
	c.2223_2231del	In-frame deletion	9	11	2	1	3	0	2	0
	c.793C>T	p.Arg265Cys	4	11	1	1	2	0	1	0
	c.350C>T	Thr117Met	9	10	1	0	4	0	3	0

	Total:		86	172	23	21	35	27	18	7

*MSH2*	c.1216C>T	p.Arg406X	5	5	2	0	1	1	0	0
	c.1165C>T	p.Arg389X	3	7	1	2	1	1	1	1
	c.1277_1386del	Exon 8 deletion	5	18	4	3	1	2	1	0
	c.2075G>T	p.Gly692Val	5	10	1	3	0	0	0	0
	c.136_164del	p.His46GlyfsX35	3	5	1	2	2	0	2	0
	c.2135_2136insT	p.Val712ValfsX4	5	11	2	1	1	0	0	0
	c.942+3A>T	Splice-site defect	13	33	3	1	1	3	1	0
	c.1511-2A>G	Splice-site defect	9	3	1	0	2	1	2	1
	c.363T>G	p.Tyr121X	10	23	3	1	7	1	1	0
	c.1_1386del	Exons 1–8 deletion	9	21	3	0	4	6	3	3
	c.1705_1706del	p.Glu569IlefsX2	3	7	1	2	1	4	1	3
	c.965G>T	p.Gly322Asp	6	14	1	0	1	0	0	0
	c.942+3A>T	Splice-site defect	5	9	2	1	2	1	2	1
	c.645+1G>A	Splice-site defect	5	7	1	2	1	0	1	0
	c.1705_1706del	p.Glu569IlefsX2	5	12	2	0	2	0	0	0
	c.1165C>T	p.Arg389X	7	22	3	1	3	6	2	3
	c.1165C>T	p.Arg389X	3	5	1	1	1	2	1	2

	Total:		101	212	32	20	31	28	18	14

*MSH6*	c.3335_3336insATGA	p.Asp1112GlufsX2	12	37	1	0	5	1	2	1

	Total:		12	37	1	0	5	1	2	1

	Grand total:		199	421	56	41	71	56	38	22

(1^st ^= first degree relatives, 2^nd ^= second degree relatives)

The Ethic approval was obtained from Mount Sinai Hospital Research Ethics Board for this research project.

### Verification of cancer diagnoses in relatives carrying a MMR gene mutation

The pathology and date of diagnosis of all subject-reported CRC in a first- or second-degree relative were verified where possible using pathology reports obtained from the OCR, other cancer registries outside of Ontario, hospital discharge data, death certificates, and reports from Regional Cancer Centres and Princess Margaret Hospital, the main cancer hospital in Toronto. If only a death certificate was available, it was reviewed. We attempted to verify cancer diagnoses reported among relatives living in other countries by requesting a pathology report from the relevant cancer registry or hospital. All probands had their colorectal cancer diagnosis confirmed by pathology reports (100%), as required for study eligibility. Of the 622 first- and second-degree relatives identified in the 32 mutation positive families, 61% of CRCs identified were confirmed by medical records and 38% of relatives identified as deceased were confirmed by death records found in the mortality database.

### Identification of probands/families carrying a MMR gene mutation

#### Microsatellite instability (MSI) analysis

Written informed consent was obtained from 99.6% of OFCCR participants for the collection of tumour tissue for use in cancer research. Colorectal tumour blocks were collected from all eligible patients. Tumour DNA was extracted from paraffin embedded matched normal and tumour tissue specimens and tested for MSI status, using a panel of 5–10 microsatellite markers recommended by the National Institute of Health [11]. MSI was defined as the presence of altered/additional bands in the polymerase chain reaction (PCR) amplified product of tumour DNA in comparison with the matched normal DNA samples obtained from the adjacent normal colon. Tumours were designated MSI-high if ≥ 40% of the markers show altered band patterns, MSI-low if there is < 40% instability, and MSS if there was no instability [11].

#### MMR gene mutation analysis

MMR (*MSH2, MLH1, MSH6*) mutational analysis was performed on DNA from peripheral blood lymphocytes or lymphoblastoid cell lines. Large genomic deletions/duplications in *MSH2 *and *MLH1 *genes were identified by MLPA [12] and, if absent, underlying germline mutations were further screened by genomic DNA sequencing [9]. Briefly, the entire coding regions of the *MSH2 *(16 exons) and *MLH1 *(19 exons) genes were amplified by PCR and screened for mutations using ABI 377 automated sequencer. Similar functional analysis was also performed for *MSH6 *gene using exon-by-exon sequencing strategy. Functional mutations were confirmed by assessing published literature, as well as from the human genome mutation database [13] and the International Collaborative Group-HNPCC database (InSIGHT). We used the computational programs Polymorphism Phenotyping to predict the pathogenicity of novel MMR alterations [14] and Sorting Intolerant From Tolerant [15]. The description of the mutations found in this study is given in Table 1.

## Statistical Methods

### Kaplan-Meier survival analysis

For the cohort of proband's family members, Kaplan-Meier survival analysis [10] was used to estimate the age-specific cumulative CRC incidence, with the corresponding 95% confidence interval (CI), where observation time was taken from birth to the earlier of age at diagnosis of CRC or current age. The analysis was stratified by: (a) MMR mutation status (*MSH2 *or *MLH1*); (b) gender among MMR mutation carriers and non-carriers.

### Segregation-based analysis

To account for missing genotype data and the non-random ascertainment of the families, we fitted likelihood-based segregation analyses [16,17]. This allowed the estimation of the cumulative risk (penetrance) associated with either any Lynch Syndrome mutation or specific *MLH1 *or *MSH2 *mutations, in males and females. Based on preliminary data analysis, we found that a log-logistic regression model for the hazard function (see Equation (1) in Appendix 1) fitted the data well, and we used this to estimate the cumulative risk of developing CRC by each decade of age, for each type of mutation. The log-logistic model had the added advantage that it allowed the hazard rate to follow a non-monotonic function, in which the risk initially increased with age and then decreased, as observed in Jenkins et al. [18].

The analyses were adjusted for ascertainment by conditioning the likelihood of the family's observed genotypes on the observed phenotypes in the family members, given their ages at examination (see Appendix 2). Thus, we obtain the maximum likelihood estimates of the needed parameters by maximizing this ascertainment-corrected retrospective likelihood [16,17] into the software Mendel [19] and used the parameters to estimate the cumulative risk of CRC at a given age, using the penetrance function in Equation (2) of the Appendix 1. For this analysis, the ascertainment correction was based on the first degree relatives of probands (i.e., the proband, his/her sibs and their parents), where probands carried a MMR mutation. For all analyses, the 95% CI for the cumulative risk by a given age was estimated using simulation of 1,000 sets of parameters, assuming a multivariate normal distribution of the parameters being estimated [18].

## Results

### Distribution of missing ages and ages at onset in the families

We had information on age at CRC onset for all 32 probands. This information was missing for 6 out of 97 affected relatives but 3 of them had their age at examination available. Among the 493 unaffected individuals (255 males and 238 females), age at examination was missing for 113 (60 males and 53 females). Among affected individuals (probands and relatives), the mean age at onset was 45.1 (n = 25, standard deviation (s.d.) = 13.3) in *MSH2 *carriers, 53.6 (n = 21, s.d. = 12.3) among *MLH1 *carriers and 51.4 (n = 76, s.d. = 14.6) for individuals with unknown mutation status. Only one individual was negative for *MSH2 *among the affected relatives.

### Kaplan-Meier analysis

Descriptive analyses with the Kaplan-Meier (KM) estimator were carried out on the sample of kin with blood and age information available. The kin included 60 individuals who were positive for any MMR mutation (16 affected and 44 unaffected individuals) and 62 who were negative (1 affected and 61 unaffected individuals). In addition, we also estimated separately the cumulative risk in 352 individuals with unknown mutation status (77 affected and 275 unaffected individuals). By age 70, about two thirds of male carriers and one half of female carriers of any MMR mutation had experienced CRC (Table 2). Importantly, the risk among those with unknown mutation status was considerably closer to that of carriers than non-carriers between the two estimates (Table 2). Thus, omitting the unknown group or combining their estimates with another genotype category could lead to serious biases.

**Table 2:** Estimates of cumulative risks (%) to ages 30, 50, and 70 (and corresponding 95% confidence intervals) for colorectal cancer stratified by MMR mutation status and gender using Kaplan-Meier analysis

**Mutation**	**Gender**	**Age 30**	**Age 50**	**Age 70**
Carrier of any MMR mutation Carrier	Male (n = 27)	-	21 (0, 39)	67 (3, 89)
	Female (n = 33)	4 (0, 10)	35 (6, 55)	51 (16, 71)
	Combined (n = 60)	2 (0, 6)	29 (10, 44)	55 (28, 72)

*MLH1 *carrier	Male (n = 13)	-	40 (0, 70)	70 (0, 94)
	Female (n = 12)	-	-	38 (0, 68)
	Combined (n = 25)	-	16 (0, 34)	50 (4, 73)

*MSH2 *carrier	Male (n = 12)	-	-	-
	Female (n = 23)	5 (0, 15)	61 (9, 83)	61 (9, 83)
	Combined (n = 35)	3 (0, 10)	36 (6, 56)	56 (15, 77)

Non-carrier of all mutations	Male (n = 22)	-	-	-
	Female (n = 40)	-	7 (0, 19)	7 (0, 19)
	Combined (n = 62)	-	4 (0, 11)	4 (0, 11)

Any mutation unknown	Male (n = 195)	2 (0, 5)	23 (14, 31)	46 (34, 57)
	Female (n = 157)	4 (0, 7)	13 (6, 20)	41 (27, 52)
	Combined (n = 352)	3 (1, 5)	19 (13, 24)	44 (35, 52)

(**n **represents the number of kin with blood available and age information available)

### Modified segregation-based analysis

The modified segregation-based approach was able to use the full cohort of probands and their kin, with or without blood available. After removing individuals without information on age at diagnosis or examination, the final sample included 506 individuals out of the 622 recruited. Therefore, the estimation of the cumulative risk with the segregation approach took into account 32 probands, 60 kin who tested positive for any MMR mutation, 62 kin who tested negative, and 352 kin with unknown mutation status. These 32 probands and 352 kin could not be included in the KM estimation of carriers and non-carriers.

**Figure 1:**
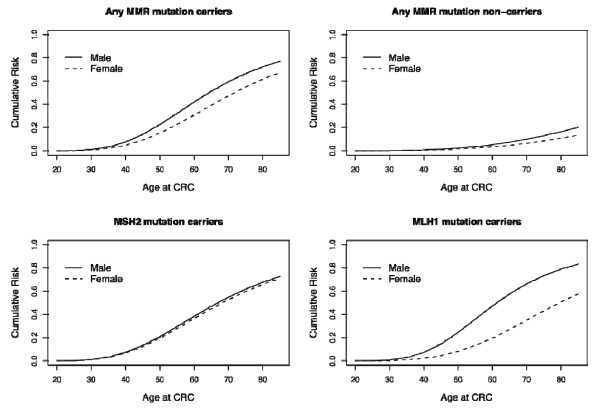
**Age-specific cumulative risks of colorectal cancer (CRC) among carriers and non-carriers of MMR (any MMR, *MLH1*, *MSH2*) mutation specified by gender, based on the segregation analysis**.

As assessed by segregation analysis, 60% of male and 47% of female carriers of any MMR gene mutation had developed CRC by age 70. These risks were 81% and 72% by age 90, respectively. The cumulative risk appeared higher for men than for women at all ages (Table 3 and Figure 1), although a formal test of difference was not significant (Wald Chi-square test; p-value = 0.25). This arose because male carriers of *MLH1 *gene mutations had a significantly higher risk of cancer than female carriers at all ages (67% vs. 35% by age 70, p-value = 0.02) (Table 3 and Figure 1), while male and female carriers of *MSH2 *mutations had similar cumulative risks of disease (≈ 54%, p-value = 0.89) (Table 3). Among men, the estimated risk was higher among carriers of *MLH1 *mutations than *MSH2 *mutations (e.g. 67% vs. 55% by age 70), while the opposite was observed among women (e.g. 35% vs. 53% by age 70).

**Table 3:** Estimates of cumulative risks (%) at ages 30, 50 and 70 (and corresponding 95% confidence intervals) for colorectal cancer among mutation carriers and non-carriers (any MMR, *MLH1*, *MSH2*) based on the segregation analysis.

**Mutation**	**Gender**	**Age 30**	**Age 50**	**Age 70**
Carrier of any MMR mutation	Male	1 (0, 2)	23 (10, 33)	60 (35, 73)
	Female	1 (0, 1)	15 (7, 23)	47(27, 60)
	Combined	1 (0, 1)	18 (11, 25)	53 (37, 64)

*MLH1 *Carrier	Male	1 (0, 2)	25 (7, 51)	67 (27, 89)
	Female	0 (0, 1)	8 (2, 19)	35 (10, 59)
	Combined	0 (0, 12)	13 (4, 30)	44 (19, 70)

*MSH2 *Carrier	Male	1 (0, 2)	21 (1, 36)	55 (2, 75)
	Female	1 (0, 2)	20 (1, 32)	53 (2, 70)
	Combined	1 (0, 2)	20 (1, 29)	54 (3, 69)

Non-carrier of all mutations	Male	0 (0, 1)	2 (0, 7)	10 (1, 27)
	Female	0 (0, 1)	1 (0, 6)	6 (1, 24)
	Combined	0 (0, 1)	2 (0, 6)	9 (2, 24)

**Figure 2:**
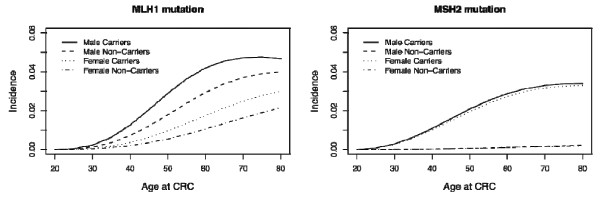
**Gender-specific and/or mutation-specific hazard rate estimates of developing colorectal cancer (CRC) in *MLH1 *gene (left) and *MSH2 *gene (right) mutations, based on the segregation analysis**.

For *MLH1 *mutations, the age-specific risk of cancer among male carriers (the hazard rate) increased to about age 70 but remained relatively constant thereafter (Figure 2), while female carriers continued to experience an increase in risk after age 70. However, for *MSH2 *mutation carriers of both sexes, risk increased only until about age 70 and stabilized afterwards. As observed in previous studies, male carriers were at higher risk than female carriers for *MLH1 *but not for *MSH2 *mutations.

Estimates of hazard ratios (HRs) for CRC at different ages are presented in Table 4. Estimates for males and females were combined in the table due to their large confidence intervals. The effect of *MLH1 *on CRC was almost constant with age (the HR varied between 5.5 at age 30 and 5.1 at age 70). *MSH2 *had a stronger effect on CRC and exhibited a decreasing pattern with age (the HR fell from 13.1 at age 30 to 5.4 at age 70). The global effect of any MMR mutation on CRC was significant and showed a decreasing trend with age (the HR fell from 10.5 at age 30 to 5.5 at age 70).

**Table 4:** Estimated hazard ratios of colorectal cancer risk at ages 30, 50 and 70 (and corresponding 95% confidence interval) in gene mutation carriers (any MMR, *MLH1*, *MSH2*) compared with that in the general population, based on the segregation analysis.

**Mutation**	**Age 30**	**Age 50**	**Age 70**
Any MMR	10.5 (2.5, 46.9)	8.8 (2.3, 40.1)	5.5 (1.8, 25.0)
*MLH1*	5.5 (0.8, 53.6)	6.5 (1.4, 41.8)	5.1 (1.3, 26.9)
*MSH2*	13.1 (0.3, 78.8)	9.3 (0.3, 44.4)	5.4 (0.3, 24.5)

## Discussion

To our knowledge, only three other studies [6,18,20] have provided population-based estimate of CRC risk in Lynch Syndrome families but none of them was reported in North America. While our results confirmed a relatively high penetrance associated with MMR gene mutations, our risk estimates seem lower than many clinic-based estimates [3,5,21-26].

Previous studies have estimated the risk of developing CRC among MMR gene mutation carriers in Lynch Syndrome families to vary between 30 to 100% [3-6,21-26], where the lowest rates are generally reported in women from population-based studies. The excess of risk in males compared to females found in several studies [3,4,6] was also confirmed in our analyses. This could suggest that females are protected from CRC; perhaps due to environmental/reproductive factors unique to women or to a sex-linked modifier gene. We also found a gender specific mutation effect with a higher risk in male carriers of *MLH1 *mutations (67%) vs. carriers of *MSH2 *mutations (55%), while the opposite was observed among women carriers (35% for *MLH1 *vs. 53% for *MSH2*), by age 70. Our study also showed that age-specific risk (hazard ratio) associated with *MLH1 *was almost constant with age, while the risk associated with *MSH2 *decreased with age. The same trends were also suggested in a recent study [18]. If differences in risks observed for *MLH1 *and *MSH2 *are confirmed, then the distribution of the types of MMR mutations could have a profound impact on the cancer risk.

Our cumulative risk estimates are close to the lower estimates previously published. They are slightly higher than another recent population-based study [18], but this latter selected exclusively early-onset probands. Several factors might explain the discrepancies with the previous penetrance estimates. First, methods of kindred ascertainment varied between studies, and the distribution of factors which likely affect risk may also vary across studies conducted in different countries. Because MMR mutations are rare in the general population [6,8], most penetrance estimates are derived from high-risk Lynch Syndrome families, who usually satisfy the original or revised Amsterdam criteria [27]. Because these designs are enriched with mutation carriers, they could be more efficient for estimating penetrance than population-based designs [17] but also more prone to biases [28,29]. Extrapolation to the general population (i.e. all CRC cases) is not possible unless appropriate ascertainment correction is applied to account for the nonrandom sampling. This was performed in this study by the use of the modified segregation-based analysis. Our recent simulation studies [17] confirmed the validity of our ascertainment-corrected approach. Second, it is likely that there exist other genetic and non-genetic contributors to HNPCC, other than a MMR mutation, that could also aggregate within families. Some of our additional analyses suggest the role of a second major gene within these families, but further work is still needed to distinguish its effect from a common environmental factor. Third, data quality was improved in the present investigation compared to previous studies in two ways. Previous studies, unlike the present study, were conducted before techniques were available to test for MMR gene mutations so could not determine accurately the status of the MMR gene [22,23]. In addition, missing genotype is a common problem in family studies and the classical analysis approaches for time-to-onset data such as Kaplan-Meier estimation or Cox regression model, in their original formulations, cannot solve this problem. In this study, the use of a modified segregation-based approach allows inferences on the missing genotypes by using the Mendelian transmission probabilities and genealogical relationships. As a consequence, the segregation-based analysis was able to use the information on 32 probands and 352 kin who were not included in the KM analyses, resulting in more precise and potentially less biased penetrance estimates.

Our estimated cumulative risks among non-carriers are much higher than observed in the general population, for example we estimated a combined (*MLH1/MSH2*) cumulative risk of 9% by age 70 while it is about 2% in the US population. The possible discrepancy between our cumulative risk estimates in non-carriers vs. those published in the US general population could be due to the fact that our sample is enriched with affected individuals who could have a very different genetic and non-genetic risk profile than the general population. Therefore, even if our design and ascertainment correction approach tend to yield estimates that are closer to the general population, the difference seen between the two estimates can reflect a difference in the distribution of risk factors in our sample compared to the general population [28].

In summary, many sources of bias have been reduced in this study, in part through the choice of study subjects and use of the modified segregation-based analyses. However, several limitations may still exist. First, the sample size is relatively small for a risk estimation study and the confidence intervals are large, especially for estimating mutation-specific and gender-specific penetrances. This problem was only partly overcome by using the modified segregation-based approach. Efficiency was also improved by selecting preferentially probands carrying a mutation and thus they are more informative than random probands [17]. Second, inaccuracy of cancer history might introduce error. While we attempted to confirm with pathology reports all reported family members with a CRC diagnosis, this was not possible for all cases. However, recent research conducted in Ontario found that proband's reports of relatives' cancer diagnoses are fairly accurate; 93% of proband-reported CRC among first-degree relatives were verified by either hospital records, cancer registry or death certificates, though reporting was less accurate for second-degree relatives with only 72% of reported CRCs verified [30]. Third, some studies classified tumours as MSI-high if > 30% of the markers show altered band patterns. Because we identified MSI-high tumours as those with altered band patterns in > 40% of markers, we may have missed some carriers. However, because this classification was not associated with the proband's family history, it is unlikely that this biased our penetrance estimates. Finally, we assumed that the probands selected are representative of the entire population of CRC families in Ontario. Because of our relatively small sample size, this hypothesis is difficult to assess and deviation from this hypothesis could lead to a selection bias. Although unlikely, it is possible that the estimates of penetrance could be biased upwards if families carrying the gene participated differentially according to the prevalence of cancer in the family.

In conclusion, this study provides a unique population-based study of CRC risks among *MSH2*/*MLH1 *mutation carriers in a Canadian population and can help to better define and understand the patterns of risks among members of Lynch Syndrome families.

## Competing interests

The authors declare that they have no competing interests.

## Authors' contributions

YC, LB performed the statistical developments and analyses and drafted the manuscript. MC conceived of the study, participated in its design and coordination and contributed to the manuscript drafting. BP carried out the molecular genetic studies. MN contributed to the statistical analysis. KB participated in the study coordination and data collection. SG, JML, GME, MA participated in the study design and manuscript drafting. All authors read and approved the final manuscript.

## Appendix 1: Log-logistic regression model

We consider a log-logistic regression model for investigating the dependence of age at onset of colorectal cancer on mutation carrier status and gender.

Suppose *T *is the age of onset associated with genotype *G *and other covariates *x*. We assume *T *follows the log-logistic distribution, which has hazard function *h*(*t*|*G*) of the form by

(1)

where *x*_1 _indicates the mutation carrier status for the MMR gene (either *MLH1*, *MSH2*, or any) and *x*_*s *_distinguishes between males (*x*_*s *_= 1) and females (*x*_*s *_= 2). Here, the parameter λ > 0 is a scale parameter and ρ > 0 is a shape parameter, which allows a non-monotonic hazard function: when ρ > 1, the hazard function is unimodal and when ρ ≤ 1, it decreases monotonically.

Then, the corresponding penetrance function is defined by the cumulative risk of a disease up to age *t *given the genotype *G *and other covariates *x*, as

(2)

## Appendix 2: Ascertainment-corrected retrospective likelihood

Our risk estimates (relative or cumulative) are based on an ascertainment-corrected retrospective likelihood approach. Correction was based on the conditional probability of the observed genotypes (*G*) given the observed phenotypes (*D*) in the family and corrected for the ascertainment event (*A*).

The ascertainment-corrected retrospective likelihood, *L*, arising from a sample of *n *independent families for each family size n_f _has the form



where *L*_*f *_is the conditional likelihood of family *f *obtained by dividing the likelihood contribution of family *f*, *N*_*f*_, by the probability of its being ascertained, *A*_*f*_.

For family *f *with *n*_*f *_family members, we define *D = (D*_1_, ..., *D*_*nf*_*) *and *G = (G*_1_, ..., *G*_*nf*_*) *as the vector forms that represent their phenotypes and genotypes, respectively, where the phenotype is defined by the age at onset and affection status, i.e. *D*_*i *_= *(T*_*i*_, *δ*_*i*_*)*. The likelihood contribution for family *f *can be expressed as a function of the genotypes of family members given their phenotypes and ascertainment event, i.e.,



Here, *P *(*D*|*G) *in the numerator can be obtained using survival analysis approach,



where *h*(.) and *S*(.) represent hazard and survivor functions, respectively, defined in Appendix 1, P(G) is the genotype probability of family members based on Hardy-Weinberg equilibrium and Mendelian transmission probabilities, and the denominator, P(D, A), represents the probability of observing the phenotypes of individuals who qualify for ascertainment. For more details, refer Choi et al. [17].
